# A biopotential optrode array: operation principles and simulations

**DOI:** 10.1038/s41598-018-20182-x

**Published:** 2018-02-09

**Authors:** Amr Al Abed, Hrishikesh Srinivas, Josiah Firth, François Ladouceur, Nigel H. Lovell, Leonardo Silvestri

**Affiliations:** 1 Graduate School of Biomedical Engineering, UNSW, Sydney, 2052 Australia; 2 School of Electrical Engineering and Telecommunications, UNSW, Sydney, 2052 Australia

## Abstract

We propose an optical electrode ’optrode’ sensor array for biopotential measurements. The transduction mechanism is based on deformed helix ferroelectric liquid crystals which realign, altering the optrode’s light reflectance properties, relative to applied potential fields of biological cells and tissue. A computational model of extracellular potential recording by the optrode including the electro-optical transduction mechanism is presented, using a combination of time-domain and frequency-domain finite element analysis. Simulations indicate that the device has appropriate temporal response to faithfully transduce neuronal spikes, and spatial resolution to capture impulse propagation along a single neuron. These simulations contribute to the development of multi-channel optrode arrays for spatio-temporal mapping of electric events in excitable biological tissue.

## Introduction

Multielectrode arrays (MEAs) are a powerful tool for recording biopotentials from excitable cells and tissue. They have been applied in electrophysiology research to map the sequential electric activation of cardiac tissue^1^ as well as neural cells and networks^2^, to study the spatio-temporal evolution of synaptic connections^3^, and in pharmacological and toxicological studies to assess the effect of chemicals on impulse excitation and propagation^4,5^.

With the growing need for higher-resolution spatio-temporal mapping of electrical excitation in neural circuits, cardiac fibres, as well as subcellular structures, there is an ongoing research effort to improve the characteristics (sensing area, electrode density, channel count, transduction/processing circuitry, longevity) and performance (speed, accuracy, signal-to-noise ratio) of MEAs. In particular, the total number of electrodes, their size and their density are limited by the complexity of interface wiring and electronics^2,6^. Smaller electrode areas allow higher density and channel count thereby enabling addressing of a larger number of cells, however leading to greater electrode-electrolyte bilayer impedance, thus signal attenuation and noise^7^. To address some of these issues, Complementary Metal Oxide Semiconductor (CMOS) technology has been employed in MEA fabrication^8–11^. In addition, carbon nanotubes and gold nanostructures have been applied to improve cell-electrode interfacing^6,11,12^ and alternative materials have been developed as transducers in MEAs, such as organic electrochemical and organic field-effect transistors^13–15^.

We propose optical biopotential transducers as an alternative approach, by replacing traditional electrodes with liquid crystal-based optical electrodes (optrodes). Our work on deformed helix ferroelectric liquid crystals (LCs) has shown that this class of materials is sensitive to very small electric fields and exhibits a fast, highly linear electro-optical response and time constant^16,17^ appropriate to be applied for measurements of biopotentials. Our approach improves the signal-to-noise ratio by optically decoupling the electrodes from both amplifiers and analog-to-digital conversion hardware in traditional MEAs. This optical decoupling confers a further advantage over CMOS MEAs in that there is no need for the electrical circuitry associated with reading and amplifying each channel to be on-chip close to the sensing electrodes. This improves spatial resolution and channel count by removing conductive tracks and chip circuitry. Theoretical impedance analysis of the device in physiological solution predicts near-unity coupling to extracellular biopotentials which is, by virtue of the scaling with area of the LC layer impedance relative to that of the bilayer, independent of optrode area.

Through computational and analytical modelling, we demonstrate the ability of an optrode array to image biopotentials by coupling them to the electrodes of a LC cell and measuring their reflectance under parallel polarisers. As a test case, we simulated biopotential signal transduction from the electrical activity of a single neuron. These results thus have important consequences for the potential development of a new class of brain-machine interfaces^18^ based on optrode arrays.

## Principle of device operation

**Figure 1:**
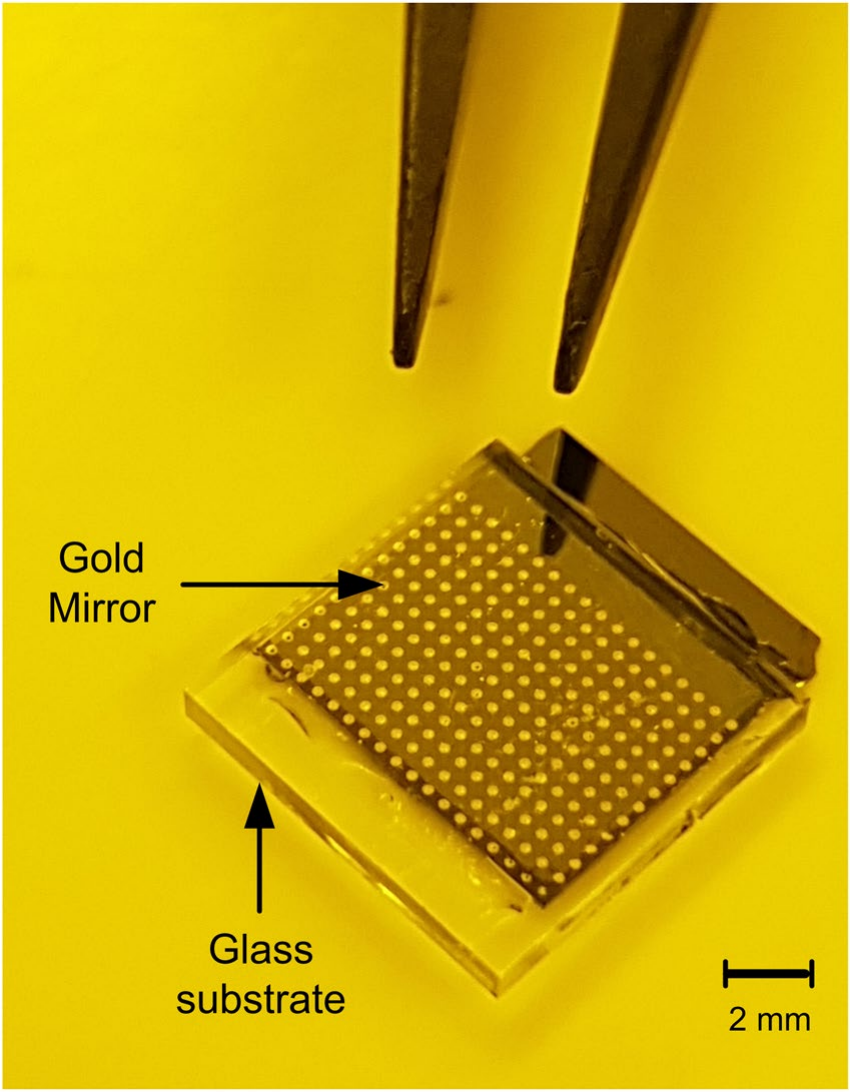
Photo of a fabricated optrode sensor array prototype, with 13 × 18 vias with centre-centre spacing of 500 *μ*m × 500 *μ*m. The gold mirrors (*ϕ* = 250 *μ*m) covering the liquid crystal side of the vias (*ϕ* = 60 *μ*m) are visible through the glass substrate, and transparent ITO and liquid crystal layers. In practice, the device will be positioned such that the glass substrate will be at the bottom and vias facing upwards in contact with the biological sample (upside down relative to this view). The overall size of this prototype is 10 mm × 11.5 mm.

**Figure 2:**
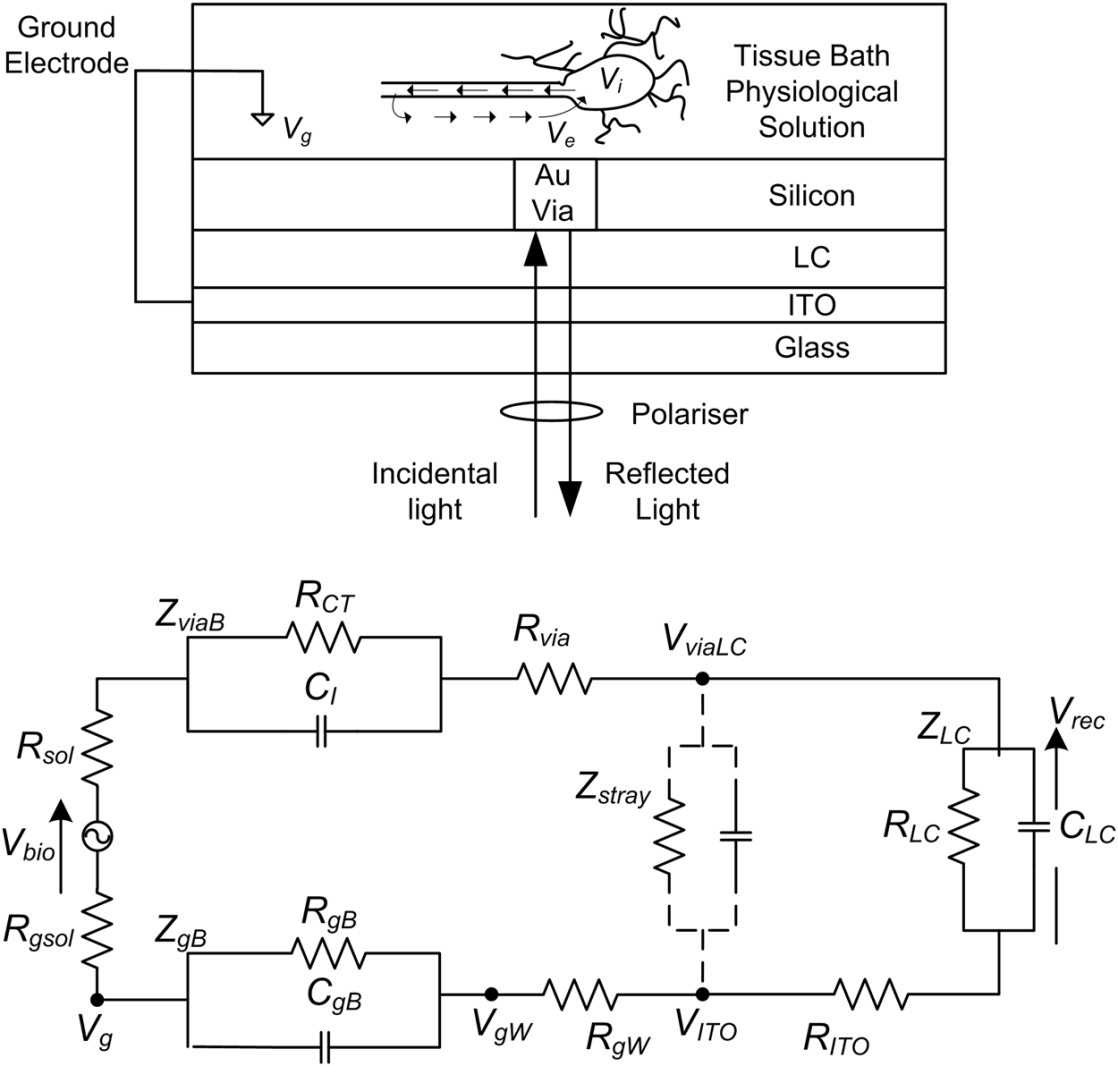
Circuit model of extracellular recording by the optical biopotential electrode (optrode). *V*_*bio*_: extracellular biopotential, *V*_*rec*_: transduced signal. *R*_*sol*_(*R*_*gsol*_): spreading or solution resistance of bathing physiological solution seen from optrode (ground). *R*_*via*_: resistance of each of the optrode vias. *R*_*gW*_: resistance of the cable connecting the ITO and ground reference electrode. *Z*_*gB*_, *R*_*gB*_, *C*_*gB*_: impedance, resistance, and capacitance of the bilayer between ground electrode and bathing solution. *R*_*gW*_: resistance of cable connecting the ITO and ground electrode. *Z*_*viaB*_, *R*_*CT*_, *C*_*I*_: impedance, resistance and capacitance of the via-bathing solution bilayer. *Z*_*stray*_: stray impedance, *Z*_*LC*_, *R*_*LC*_, *C*_*LC*_: impedance, resistance, and capacitance of the LC layer. *R*_*ITO*_: resistance of the ITO layer.

The schematic and circuit model of our optrode device (Fig. 1) are illustrated in Fig. 2. Its core is a 3 *μ*m thick LC cell sandwiched between two substrates: a silicon wafer (topside) with a grid of gold through-silicon vias, making an electrical connection between the LC at the bottom and the biological tissue above; the bottom substrate is glass with an ITO layer coated on the inner surface. The whole device constitutes the bottom of a recording chamber containing physiological solution where biological tissues are placed in close contact with the metal vias. The gold vias and the ITO layer are effectively the electrodes of the LC cell. Importantly, the ITO and the physiological solution are connected via a reference ground electrode.

When a neuron, cardiac or muscle fibre is stimulated (via synaptic inputs or artificial current injection), current flows inside the cell (intracellularly) away from the site of initial excitation. As current spreads it causes a change in the intracellular voltage. This charges up the cell membrane which acts as a capacitor, leading to a change in the local potential difference across the cell membrane. The change in the transmembrane potential opens voltage-sensitive ion channels embedded in the membrane, leading to a local movement of ions in and out of the cell. Both capacitive and ionic currents lead to a local change in the electrical potential of the extracellular space around a neuron/muscle fibre.

The principle of operation of the optrode device is as follows. The electric potential generated by the biological specimen is picked up by the vias and then applied to the LC cell, so that a small electric field is generated in the LC layer. The gold also acts as a broadband mirror, so that light shone from the bottom of the device traverses the LC layer and is reflected back at each via. Using a parallel polariser configuration at a particular angle the amount of light reflected can be made proportional to the applied voltage^17^. In this way it is possible to read the voltage at each electrode optically, without any electrical wiring tracks or amplifiers. The details of the optical reading mechanism have been already described^16^.

## Mathematical modelling

**Figure 3:**
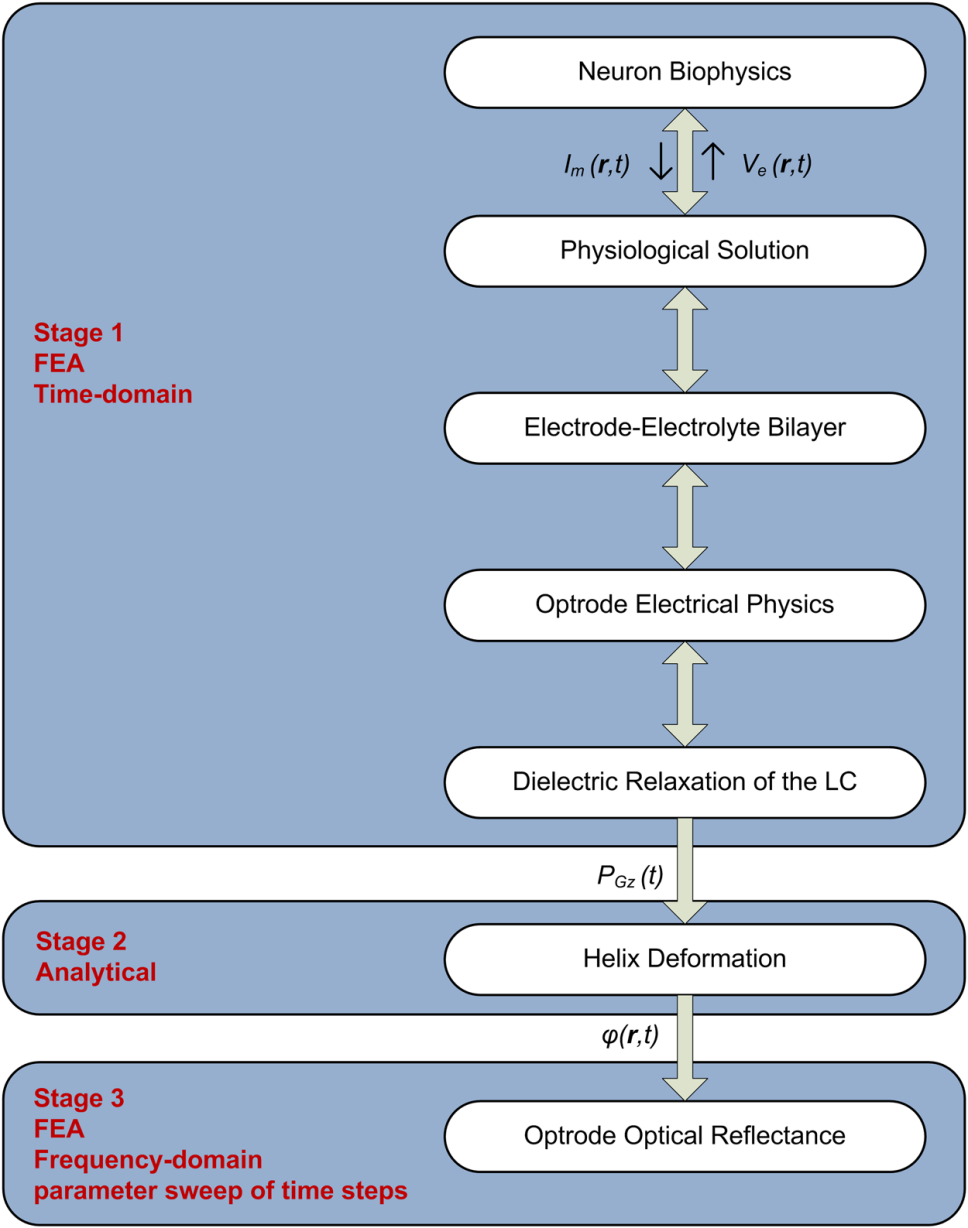
Flow chart of pipeline used to model the optrode device’s operation. Stage 1: the neuronal biophysics was fully coupled to the electrical physics of the device and physiological solution, including Debye formulations for the dielectric relaxation of the liquid crystal (LC) and electrode-electrolyte bilayers. Finite element analysis (FEA) was performed in the time-domain. Stage 2: The azimuthal angle (*φ*(**r**, *t*)) for the LC helix deformation was analytically calculated from the polarisation of the LC in the electric field direction *P*_*Gz*_(*t*). Stage 3: For each *φ*(**r**) a frequency domain FEA was used to calculate the optrode’s reflectance at each instance (*t*). By sweeping through the time points, change in reflectance with time was simulated. Note that **r** denotes the global Cartesian coordinates.

The optrode’s performance in detecting and spatio-temporal imaging of extracellular biopotentials from a single neuron was simulated using a combination of finite element and analytical methods using COMSOL Multiphysics (COMSOL AB, Sweden), Mathematica (Wolfram Research, USA), and R (R Foundation for Statistical Computing, Austria). The electrical physics and the neuronal biophysics were solved simultaneously and fed into the optical physics (Fig. 3). We assumed the light wave does not affect the dielectric properties of the LC and hence adopted one-way coupling of the LC electrical polarisation and optical physics. Unless otherwise stated, parameter values are listed in the supplementary information.

### Electric coupling and neuronal biophysics

**Figure 4:**
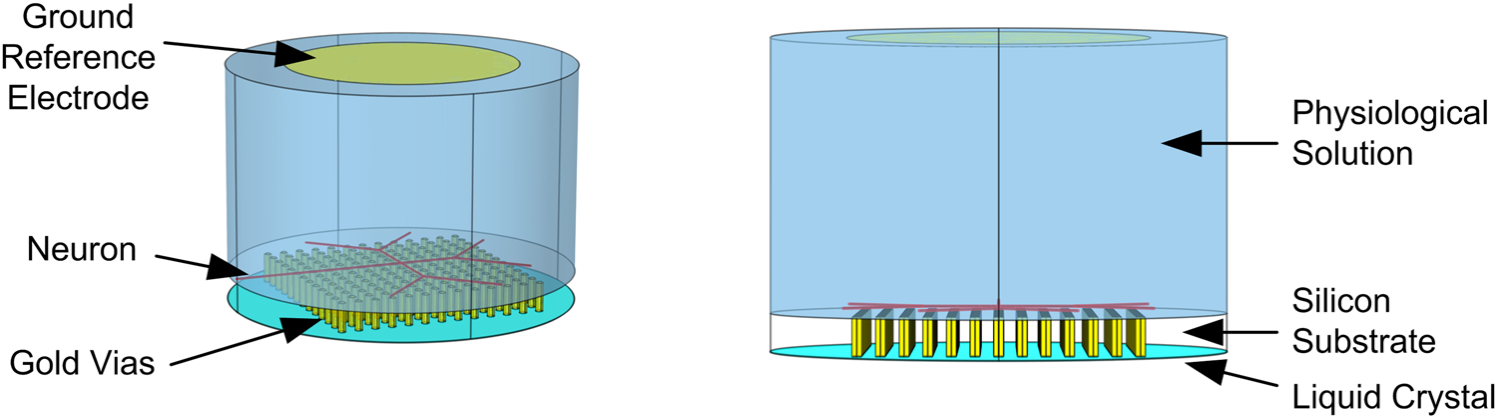
Geoemtry of the optrode device model used for time-domain finite element simulations.

The optrode device was constructed as a cylindrical object (*ϕ* = 3 mm) consisting of a LC layer (3 *μ*m) and a Si substrate (275 *μ*m) transversed with a 13 × 13 array of cylindrical gold vias (*ϕ* = 60 m) with a via centre-centre spacing of 150 *μ*m × 150 *μ*m (Fig. 4). The bottom boundary represents the ITO. A simplified geometrical representation of a neuron with two level branching was constructed in a plane parallel to and 50 *μ*m above the top plane of the vias. The neuron was immersed in a cylindrical layer of height 2 mm representing the bathing physiological solution. The ground reference electrode was defined as a disc (*ϕ* = 2 mm) at the centre of the top boundary of the physiological solution layer.

Electric excitation and propagation in the neuron were modelled using the bidomain cable formulation of the Hodgkin Huxley equations^19^

1 $$\nabla \cdot ({\sigma }_{i}\nabla {V}_{i})-\nabla \cdot (-{\sigma }_{e}\nabla {V}_{e})=\frac{2}{r}{J}_{ion}$$

2 $${V}_{m}={V}_{i}-{V}_{e}$$

where at each point on the neuron *V*_*m*_, *V*_*i*_, *V*_*e*_ are the transmembrane, intracellular, and extracellular potentials (V) respectively, with neuron radius *r*, and *σ*_*e*_ and *σ*_*i*_ the extracellular physiological solution and neuronal intracellular conductivities, respectively. *J*_*ion*_ (Am^−2^) is the ionic component of the total membrane current per unit membrane area (*J*_m_).

3 $${J}_{m}={C}_{m}\frac{d{V}_{m}}{dt}+{J}_{ion},$$

and is contributed to by time- and voltage-dependent sodium *J*_*Na*_ and potassium *J*_*K*_ fluxes, as well as a voltage-dependent leakage flux *J*_*L*_,

4 $${J}_{ion}={J}_{Na}({V}_{m},t)+{J}_{K}({V}_{m},t)+{J}_{L}({V}_{m}\mathrm{).}$$

Electric current flow in the physiological solution and the optrode’s various layers was described using the following constitutive laws:

$${\bf{J}}=\{\begin{array}{ll}(\sigma +{\varepsilon }_{0}\frac{d}{dt}){\bf{E}}+\frac{d{\bf{P}}}{dt} & {\rm{in}}\,{\rm{LC}}\,{\rm{layer}}\\ (\sigma +{\varepsilon }_{0}{\varepsilon }_{r}\frac{d}{dt}){\bf{E}} & {\rm{elsewhere}}\end{array}$$

$${\bf{E}}=-\nabla V$$

5 $$\nabla \cdot {\bf{J}}=\{\begin{array}{ll}2\pi r{J}_{m} & {\rm{on}}\,{\rm{neuron}}\,{\rm{edges}}\\ 0 & {\rm{elsewhere}}{\rm{.}}\end{array}$$

At the neuron edges *V* is equivalent to *V*_*e*_ in equations (1), (2). To model the change in the local potential produced by the neuron’s electrical activity, a line current source was added at the neuron edges.

Auxiliary equations for the polarisation **P** in the LC are presented in a later section describing the Debye dielectric relaxation of the LC material.

A Dirichlet boundary condition was imposed at the ground reference electrode (*V*_*g*_ = 0).

To calculate the voltage at the ITO boundary *V*_*ITO*_, the following equation was used to represent the ground electrode bilayer:

6 $$\begin{array}{rcl}{I}_{g} & = & {C}_{gB}\frac{d({V}_{gW}-{V}_{g})}{dt}+\frac{{V}_{gW}-{V}_{g}}{{R}_{gB}}\\  & = & \frac{{V}_{ITO}-{V}_{gW}}{{R}_{gW}},\end{array}$$

where *C*_*gB*_ and *R*_*gB*_ are the overall capacitance and resistance of the ground bilayer, obtained from equations (15) and (17) respectively, and *R*_*gW*_ is the resistance of the cable connecting the ITO and ground electrode. The current flowing through the ground electrode boundary *S* is

7 $${I}_{g}(t)={\oint }_{S}{\bf{n}}\cdot {\bf{J}}(t)\,dS\mathrm{.}$$

Finally, with a negligibly small ITO resistance, the sensed biopotential *V*_*rec*_ (V) at each optrode/channel is measured as

8 $${V}_{rec}={V}_{viaLC}-{V}_{ITO}$$

where *V*_*viaLC*_ is the electric potential at the centre of each of the via-LC boundaries.

On all other external boundaries, an insulating Neumann boundary condition was imposed:

9 $${\bf{n}}\cdot {\bf{J}}=0.$$

### Optrode-electrolyte bilayers

The boundaries between the optrode array vias and physiological solution, forming electrochemical bilayers, were modelled as contact impedances, using the thin layer simplification to overcome the need to mesh a large-aspect ratio geometrically-explicit bilayer domain^20^:

10 $${\bf{n}}\cdot {{\bf{J}}}_{s}=(\frac{1}{{\rho }_{viaB}}+{C}_{viaB}\frac{d}{dt})({V}_{s}-{V}_{via})$$

11 $${\bf{n}}\cdot {{\bf{J}}}_{via}=(\frac{1}{{\rho }_{viaB}}+{C}_{viaB}\frac{d}{dt})({V}_{via}-{V}_{s})$$

where *ρ*_*viaB*_ (Ωm^2^) and *C*_*viaB*_ (Fm^−2^) are the specific resistance and specific capacitance respectively of each via bilayer. These were obtained by theoretical consideration of the overall optrode-electrolyte impedance using the Randles circuit model^21,22^ excluding the via metal resistance *R*_*via*_, the Warburg impedance describing surface pH variation due to the biopotential, and the spreading or solution resistance for a single via of flat circular area *A* in solution of resistivity *ρ*_*sol*_,

12 $${R}_{sol}=\frac{{\rho }_{sol}}{4}\sqrt{\frac{\pi }{A}}\approx 8.3\,k{\rm{\Omega }},$$

which are small by comparison with the remaining surface impedance scaling inversely with optrode area: an interfacial capacitance shunted by a charge transfer resistance.

Electrode surface irregularities, leading to an effective surface area greater than the geometric area, were neglected in favour of a worst-case high-impedance interfacial capacitance value. This was modelled by the series combination of Helmholtz^23^ and Gouy-Chapman-Stern capacitances^24–26^ describing Helmholtz plane and diffuse layer dynamics,

13 $$\frac{1}{{C}_{viaB}}=\frac{1}{{C}_{H}}+\frac{1}{{C}_{G}}=\frac{{d}_{OHP}}{{\varepsilon }_{0}{\varepsilon }_{r}}+\frac{{L}_{D}}{{\varepsilon }_{0}{\varepsilon }_{r}\,\cosh (zV\mathrm{/2}{U}_{t})}$$

where *V* is the applied voltage at the optrode-electrolyte interface, in our case due to the sensed extracellular potential, with constants and parameters listed in supplementary information, assuming physiological saline solution of 0.154 M concentration at temperature *T* = 25 °C.

The overpotential *η* = *ϕ* − *ϕ*_0_, where *ϕ* is the potential of an electrode with current flowing between itself and a reference electrode, and *ϕ*_0_ = 1.68 V the standard reduction potential of gold referred to the standard hydrogen electrode, is here a measure of how far from equilibrium the optrode is being driven by the sensed biopotential (see for example the modelling approach of Cantrell *et al.*^20^). The Debye length of the diffuse layer, from a first order approximation valid for small signal perturbation *η* ≤ 50 mV/*z*, is

14 $${L}_{D}=\sqrt{\frac{{\varepsilon }_{0}{\varepsilon }_{r}{U}_{t}}{2{n}^{0}{z}^{2}e}}\approx 8.75\,\AA $$

giving the biopotential-dependent capacitance

15 $${C}_{viaB}={(0.5647+\frac{0.9878}{\cosh (V\mathrm{/0.0514)}})}^{-1}\,{{\rm{Fm}}}^{-2}\mathrm{.}$$

The charge transfer resistance describing Faradaic (conductive) processes across the via bilayer is given in general by the Butler-Volmer equation ignoring mass-transfer and diffusional effects^27^

16 $${\rho }_{BV}=\frac{\eta }{{J}_{0}}{(\exp (-\alpha \frac{zF}{RT}\eta )-\exp (\mathrm{(1}-\alpha )\frac{zF}{RT}\eta ))}^{-1},$$

where *J*_0_ = 20 *μA*/*m*^2^ is the equilibrium exchange current density for gold in buffered saline quoted by Najafi and Wise^28^, *α* is the reaction transfer coefficient, *R* is the ideal gas constant and *F* is the Faraday constant. Expressions for cathodic and anodic currents across the optrode surface are summed to yield, in the limiting case of small overpotential operation,

17 $${\rho }_{viaB}\approx \frac{1}{2}\frac{RT}{zF{J}_{0}}\approx 642\,{{\rm{\Omega }}m}^{2},$$

i.e. the charge transfer is independent of overpotential and ohmic.

### Dielectric relaxation of the LC

The optrode’s optical response relies on the deformed helix ferroelectric (DHF) mode of operation of ferroelectric liquid crystals (FLCs) in their smectic C* phase^29^. In this phase molecules are arranged in smectic layers, with their director changing its orientation from one layer to another. In the absence of any external electrical field, the director rotates around a cone with a fixed tilt angle, *θ*, forming a helical structure with its axis perpendicular to the smectic layers. In the simulation we use parameters typical of the LCs we used in our previous works^17,30^, with large tilt angle, *θ* = 32°, and short helix pitch, *p*_0_ = 200 nm. When electrical fields smaller than a critical value, *E*_*c*_, are applied in a direction perpendicular to the helix axis, the helical structure is deformed, but the tilt angle and the helical pitch are unchanged. In the case of biological potentials of the order of 100 *μ*V across a 3 *μ*m cell, the resulting electrical fields are much smaller than the critical value for our LCs, *E*_*c*_ = 1.56 V/*μ*m, and we can safely assume that *p*_0_ and *θ* are constant.

In the model, electrical coupling to the LC and its dielectric relaxation are included through the polarisation vector *P*. FLC molecules possess a spontaneous polarisation, *P*_*s*_, lying in the smectic layer plane and oriented perpendicular to the director, and they can give rise to a macroscopic polarisation when equilibrium is perturbed. As a consequence, the dielectric response consists of two parts: a low frequency one, connected to changes in the molecular arrangement, and a high frequency one, connected to the electronic response. Polarisation in the LC is therefore decomposed into a high frequency part, *P*_∞_, that instantaneously re-orients with the electric field, and a low frequency part, *P*_*G*_, that corresponds to deformations of the helical structure. At frequencies up to 10 kHz, the main relaxation mechanism is the Goldstone mode of the LC, which corresponds to a reorientation of the director^31^.

The auxiliary equations for the polarisation are:

18 $${\bf{P}}({\bf{r}},t)={{\bf{P}}}_{\infty }({\bf{r}},t)+{{\bf{P}}}_{G}({\bf{r}},t)$$

19 $${{\bf{P}}}_{\infty }({\bf{r}},t)={\varepsilon }_{0}({\varepsilon }_{\infty }-\mathrm{1)}{\bf{E}}({\bf{r}},t)$$

20 $${{\bf{P}}}_{G}({\bf{r}},t)=-{\tau }_{c}\frac{d{{\bf{P}}}_{G}({\bf{r}},t)}{dt}+{\varepsilon }_{0}({\varepsilon }_{s}-{\varepsilon }_{\infty }){\bf{E}}({\bf{r}},t),$$

where *τ*_*c*_ is the LC’s characteristic relaxation time, *ε*_0_ is the vacuum permittivity and *ε*_*s*_ and *ε*_∞_ are the static and high frequency LC relative permittivities, respectively^32^, and ***r*** the global Cartesian coordinates. The above equations describe Debye relaxation and are equivalent to assuming a frequency dependent relative permittivity for the LC of the form

21 $${\varepsilon }_{LC}(\omega )={\varepsilon }_{0}{\varepsilon }_{\infty }+\frac{{\varepsilon }_{0}({\varepsilon }_{s}-{\varepsilon }_{\infty })}{1+i{\tau }_{c}\omega }\mathrm{.}$$

This behaviour has been verified experimentally and the estimated values of the parameters are *ε*_*s*_ = 33.3, *ε*_∞_ = 3.5 and *τ*_*c*_ = 19.3 *μs*^32^.

### Helix deformation

The macroscopic polarisation of the LC, **P**, introduced in the previous section, is not sufficient to calculate the reflectance of an optrode. Since the LC effectively behaves as a diffraction grating, it is necessary to know how the orientation of the LC molecules changes when exposed to the electric field applied at a given via. In order to link the macroscopic electrical properties of the LC to the microscopic molecular arrangement, we provide here a complementary description of the dielectric relaxation.

The orientation at each point in space is uniquely determined by the azimuthal angle of rotation of the director in the smectic plane, *φ*, as we assumed that the tilt angle is constant. For a small uniform electric field, *E*_*z*_(*t*), along *z* and perpendicular to the helix axis *x*, the dynamic equation for *φ*(**r**, *t*) describing the relaxation due to the Goldstone mode is^33^

22 $${\tau }_{c}\frac{\partial {\varphi }}{\partial t}-{(\frac{{p}_{0}}{2\pi })}^{2}\frac{{\partial }^{2}{\varphi }}{\partial {x}^{2}}=\frac{{E}_{z}(t)}{{E}_{c}}\,\sin \,{\varphi },$$

which admits a solution of the form^33^

23 $${\varphi }({\bf{r}},t)=\frac{2\pi }{{p}_{0}}x+a(t)\,\sin [\frac{2\pi }{{p}_{0}}x],$$

with *a* ∝ *E*_*z*_/*E*_*c*_. The above solution is correct to the first order in the small parameter *a*. Since the low frequency polarisation, *P*_*G*_, is the macroscopic average of the molecular spontaneous polarisation, its *z* component is

24 $${P}_{Gz}(t)=-{P}_{s}{\int }_{0}^{{p}_{0}}\,\cos [{\varphi }(x,t)]dx/{p}_{0}\approx {P}_{s}\frac{a(t)}{2},$$

where again we used the fact that ${E}_{z}\ll {E}_{c}$ and only kept terms that are first order in *E*_*z*_. The above equation links the microscopic geometry of the LC to the macroscopic polarisation and allows us to calculate *a*(*t*) as

25 $$a(t)=2\frac{{P}_{Gz}(t)}{{P}_{s}}\mathrm{.}$$

Moreover, by comparing equations (22), (23) and (20) in the case of a constant electric field, we find the useful relation

26 $$\frac{{P}_{s}}{2{E}_{c}}={\varepsilon }_{0}({\varepsilon }_{s}-{\varepsilon }_{\infty }),$$

from which we can calculate *P*_*s*_ = 82.3 nC/cm^2^. In our simulated device the via radius is 30 *μ*m and the Gaussian light beam is focused on a spot of 10 *μ*m radius at the centre of the electrode, where we will show that the electric field is very uniform. In this region we can calculate the azimuthal angle as

27 $${\varphi }(x,t)=\frac{2\pi }{{p}_{0}}x+2\frac{{P}_{Gz}(t)}{{P}_{s}}\,\sin [\frac{2\pi }{{p}_{0}}x]\mathrm{.}$$

### Reflectance of the optrodes

A voltage change across the optrodes is detected by measuring the change in the reflectance of a light beam impinging at normal incidence from the bottom of the device through a polariser. The angle between the polariser and the LC helix axis, *β* = 30°, is thus selected in order to have a linear relationship between voltage and reflectance, as well as a large change in reflectance per applied Volt. Based on preliminary experimental work, the most accurate way of measuring such reflectance changes in practice is by using an optical fibre pigtailed to a focusing GRIN lens. This is therefore the system we simulate.

**Figure 5:**
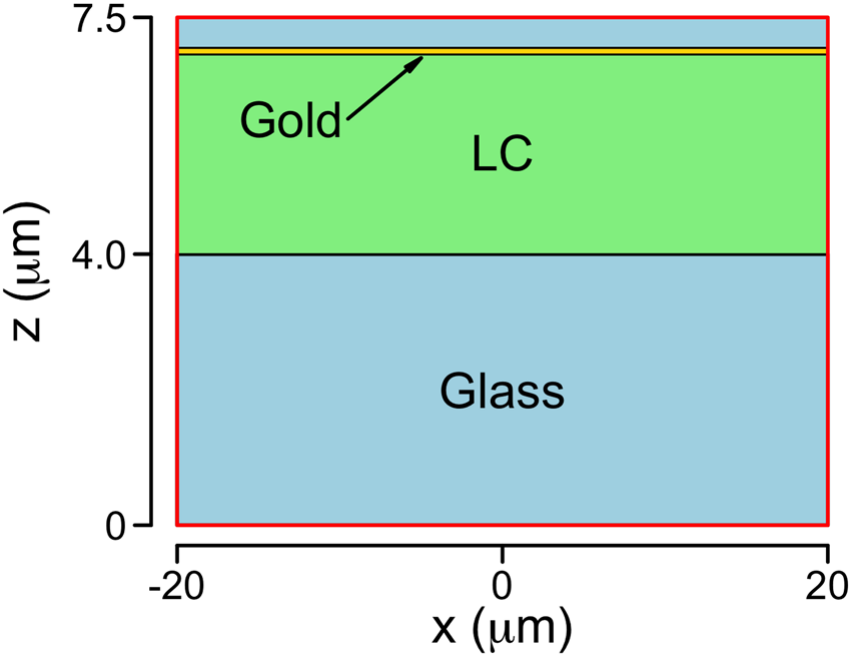
Geometry of the optrode wave optics simulations. The simulation domain is indicated by the red rectangle. Coloured regions represent the different materials (LC: liquid cyrstal). The light blue area at the top is glass, with a 60 nm thick gold layer located at *z* = 7 *μ*m.

Reflectance from the bottom of a single optrode is calculated in COMSOL using the 2D geometry shown in Fig. 5 and the “wave optics” module. The problem is solved in the frequency domain at each time step solved for in the previous part, where *P*_*G*_ was calculated. The frequency, *f* = 193414.5 GHz, is in the optical region and it is related to the wavelength of the incident light, *λ* = 1.55 *μ*m. The size of the simulation domain in the helix axis direction *x* is 40 *μ*m, large enough to fully describe the Gaussian beam. Periodic boundary conditions have been imposed at *x* = ±20 *μ*m. From bottom to top, the simulation domain includes the top of the glass substrate, described by an isotropic refractive index *n*_*g*_ = 1.52, the LC layer and the bottom of the gold optrode, described as gold coated glass in order to reduce the computational complexity. In particular, the gold coating is simulated as a “transition boundary condition” with a layer thickness of 60 nm and the appropriate wavelength dependent permittivity for the gold^34^. We have checked that, within the numerical errors, the reflectivity of such a mirror is indistinguishable from the expected value for bulk gold. More details about this boundary condition are given in the supplementary information.

The ITO layer between the glass substrate and the LC has not been included in the simulation since, for a typical optrode, its thickness is around 25 nm and it is therefore not expected to perturb the shape of the propagating Gaussian beam. On the other hand, we have experimentally measured the transmission at *λ* = 1.55 *μ*m of the ITO coating we use and we found it to be 77%. We have therefore rescaled the final reflectance to account for the corresponding losses. We believe that this procedure is better than including the ITO layer in the FEA simulation, mainly because it is difficult to find accurate microscopic parameters for specific ITO coatings, especially in the infrared region of the spectrum.

At optical frequencies the LC is birefringent and its relative permittivity tensor can be calculated as

28 $${\varepsilon }_{ij}(x,t)=({\varepsilon }_{\perp }+\varepsilon ^{\prime\prime} ){\delta }_{ij}+({\varepsilon }_{\parallel }-{\varepsilon }_{\perp }){d}_{i}(x,t){d}_{j}(x,t),$$

where *i,j = {x,y,z}* and the director is given explicitly by

29 $${\bf{d}}(x,t)=\,\cos \,\theta \,\hat{{\bf{x}}}+\,\sin \,\theta \,\cos \,{\varphi }(x,t)\,\hat{{\bf{y}}}+\,\sin \,\theta \,\sin \,{\varphi }(x,t)\,\hat{{\bf{z}}},$$

and we introduced an isotropic imaginary part *ε*′′ = 0.01*i* to account for losses in the LC due to unwanted scattering. The values of the dielectric tensor can be related to the ordinary (*n*_*o*_) and extraordinary (*n*_*e*_) refractive index as

30 $${\varepsilon }_{\perp }={n}_{o}^{2}=2.25\,;\quad \,{\varepsilon }_{\parallel }={n}_{e}^{2}=\mathrm{2.96.}$$

**Figure 6:**
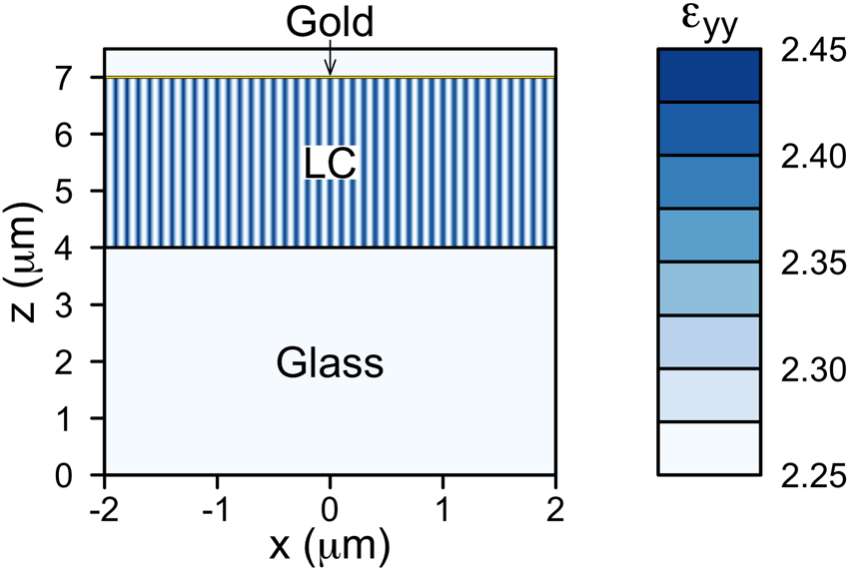
The optical permittivity of the liquid crystal (LC) is periodic along *x* with a period of 0.2 *μ*m and behaves as a diffraction grating for light polarised along the *y* axis. The value of the relative permittivity *ε*_*yy*_ (non-dimensional) at *t* = 0 ms in a small central region of the simulated domain is illustrated (colour legend on the right). The isotropic refractive index of glass is also colour-coded with the same scale for comparison. The horizontal lines represent the boundaries of the LC.

The above relations arise from the fact that LCs in the smectic phase have a nematic-like order in each smectic layer along the director. The optical permittivity of the LC is periodic in *x* with a period of *p*_0_ and behaves as a diffraction grating for light polarised along the *y* axis. As an example, the calculated *ε*_*yy*_ at *t* = 0 is reported in Fig. 6. It may be noted that the LC pitch is much shorter than the incident wavelength.

Appropriate “scattering boundary conditions” at *z* = 0 *μ*m have been introduced to simulate a linearly polarised incoming Gaussian beam with its polarisation at angle *β* to the helix axis *x*. The incident electric field was expressed as

31 $${E}_{0x}=\,\cos (\beta )g(z-{z}_{0})$$

32 $${E}_{0y}=\,\sin (\beta )g(z-{z}_{0})$$

where *z*_0_ = 7 *μ*m is the position of the beam waist focused on the gold mirror, and

33 $$g(z)=\sqrt{\frac{{w}_{0}}{w(z)}}\,\exp [-{(\frac{x}{w(z)})}^{2}]\,\exp [-i\frac{\pi {x}^{2}}{\lambda {R}_{g}(z)}+i\frac{\eta (z)}{2}]\mathrm{.}$$

The spot size parameter, *w*(*z*), the radius of curvature of the beam’s wavefront, *R*_*g*_(*z*), and the Gouy shift, *η*(*z*), are given by

34 $$w(z)={w}_{0}\sqrt{1+{(\frac{z}{{z}_{0}})}^{2}}$$

35 $${R}_{g}(z)=\frac{{z}^{2}+{z}_{0}^{2}}{z}$$

36 $$\eta (z)=\arctan (z/{z}_{0}),$$

where *w*_0_ = 10 *μ*m is the beam waist radius. The total optical power per unit length of the input wave, *I*_0_, has been calculated by integrating the Poynting vector over the *z* = 0 boundary in a simulation where the whole domain was made of glass. At each time step, the reflectance through the analyser, which coincides with the polariser and is therefore parallel to the input polarisation, can be expressed as

37 $$R(t)=1-\frac{{I}_{R}(t)}{{I}_{0}},$$

where *I*_*R*_ was calculated by projecting the electric field at *z* = 0 onto the unit polariser $\hat{{\bf{p}}}$, then integrating the *z* component of the Poynting vector over the *z* = 0 boundary

38 $${I}_{R}(t)={\int }_{-20\mu {\rm{m}}}^{20\mu {\rm{m}}}\frac{1}{2}{\rm{Re}}[({\bf{E}}(x,z=\mathrm{0,}\,t)\cdot \hat{{\bf{p}}})\hat{{\bf{p}}}\times {{\bf{H}}}^{\ast }(x,z=\mathrm{0,}\,t)]dx\mathrm{.}$$

### Data availability

All data generated or analysed during this study are included in this published article and its supplementary information files.

## Results

### Simulated device performance

**Figure 7:**
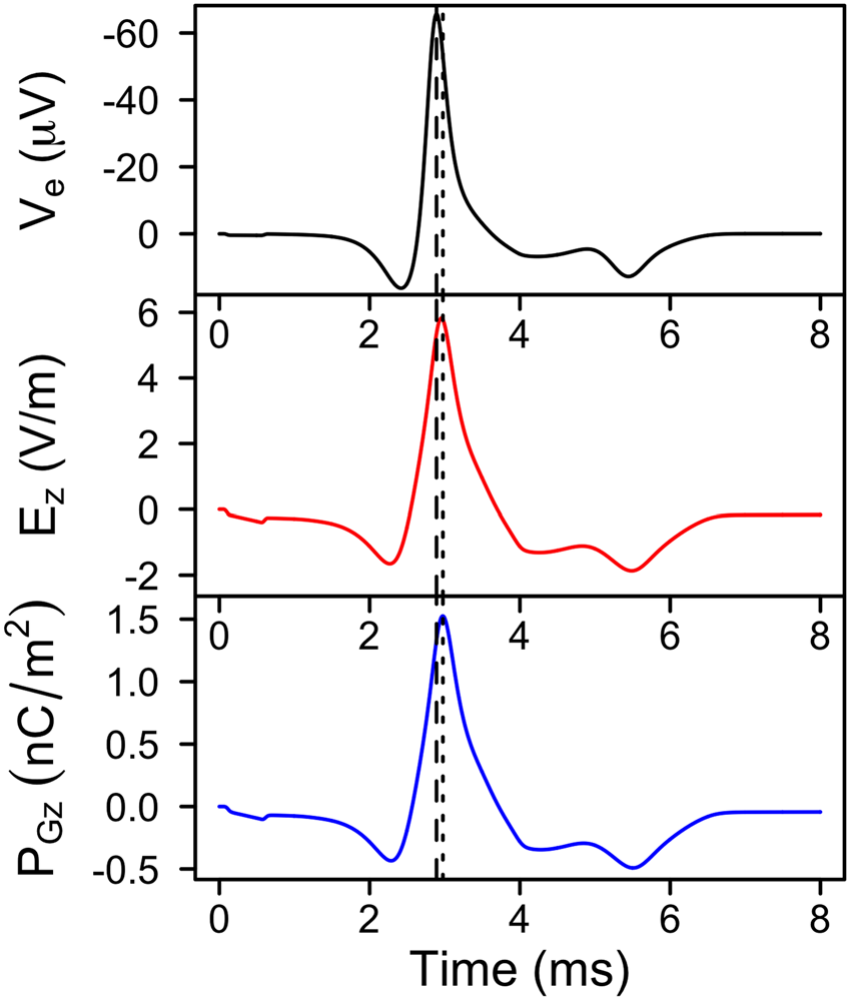
Simulated temporal performance of the optrode at a single channel. Comparison of temporal changes in extracellular potential probed at the neuron (*V*_*e*_), electric field across the liquid crystal (LC) layer (*E*_*z*_), and electric polarisation of the LC (*P*_*Gz*_) in the direction perpendicular to the optrode’s circular surface, measured directly below the neuron probe point.

To assess the temporal performance of the optrode’s electro-optical transduction mechanism, we simulated a single channel optrode device with refined mesh and time-stepping routines. The extracellular voltage trace measured at the neuron correlates well with the reflectance of the channel directly below, with a phase shift of 15 *μ*s between the peak reflectance and the (negative) peak of extracellular potential spike. This shift is solely attributed to the LC polarisation, as shown in Fig. 7, and is well within the Debye relaxation time constant of the LC.

**Figure 8:**
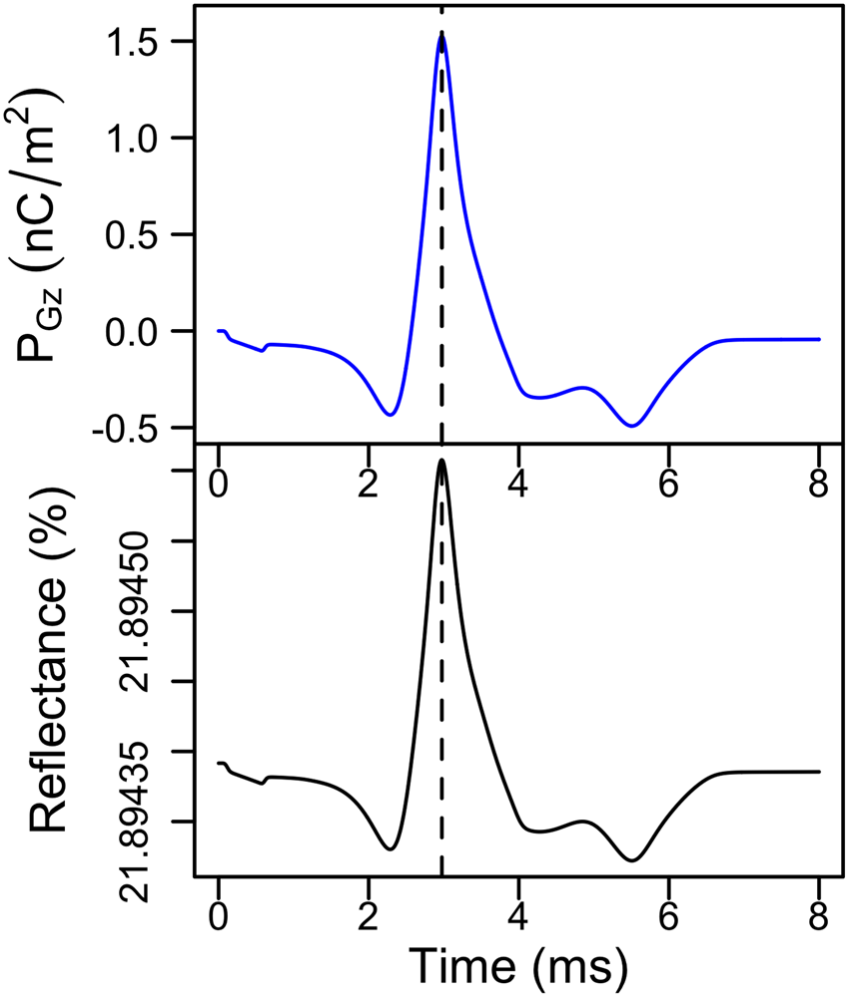
Simulated temporal performance of the optrode at a single channel. Comparison of temporal changes in electric polarisation of the LC (*P*_*Gz*_) in the direction perpendicular to the optrode’s circular surface, and reflectance *R* under parallel polariser/analyser. *P*_*Gz*_ was measured directly below the neuron probe point.

**Figure 9:**
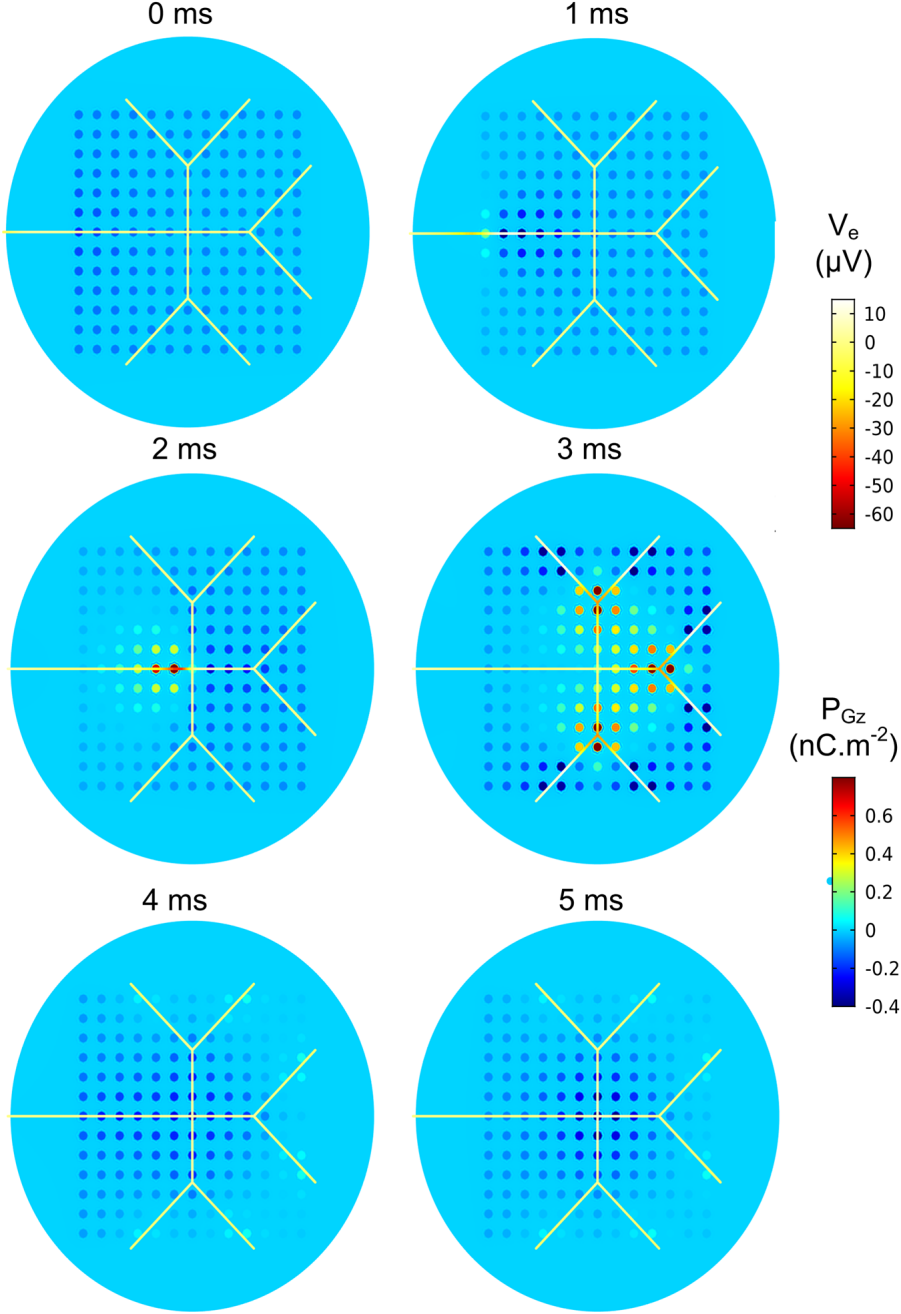
Simulated spatial performance of a 169 channel optrode device. Cross-sectional view of the optrode array at different time points relative to the endpoint of the electrical current pulse used to excite the leftmost segment of the neuron. The extracellular potential at the neuron, *V*_*e*_, is overlaid on the electric polarisation of the liquid crystal in the direction perpendicular to the plane of sensing, *P*_*Gz*_. The neuron diameter is not drawn to scale.

As no phase shift was measured between reflectance and LC polarisation (Fig. 8), the temporal changes in the latter could be used as a surrogate of the former. This permits reconstructing spatial activation maps without the need to run parametric sweeps and frequency domain based FEA (Fig. 3) in order to calculate the reflectance at every single optrode in the 169 channel modelled device (see Fig. 9).

Spatial activation maps from the optrode array simulation output indicate that the neural impulse propagates at a speed of 0.77 ms^−1^ as measured between optrodes (1, 7) and (10, 7), which is similar to the neuron’s impulse conduction velocity of 0.78 ms^−1^.

### Electrical analysis of device

The relative change in *E*_*z*_ along a 40 *μm* line segment midway through the LC, below and parallel to the via-LC interface, was quantified to be 3 × 10^−6^. This change is significantly smaller than the relative tolerance of the numerical solver, 0.01, hence we can assume that *E*_*z*_ is effectively uniform in the central 40 *μm* region of each channel, justifying our formulations for helix deformation and optical reflectance in equations (22)–(27).

**Figure 10:**
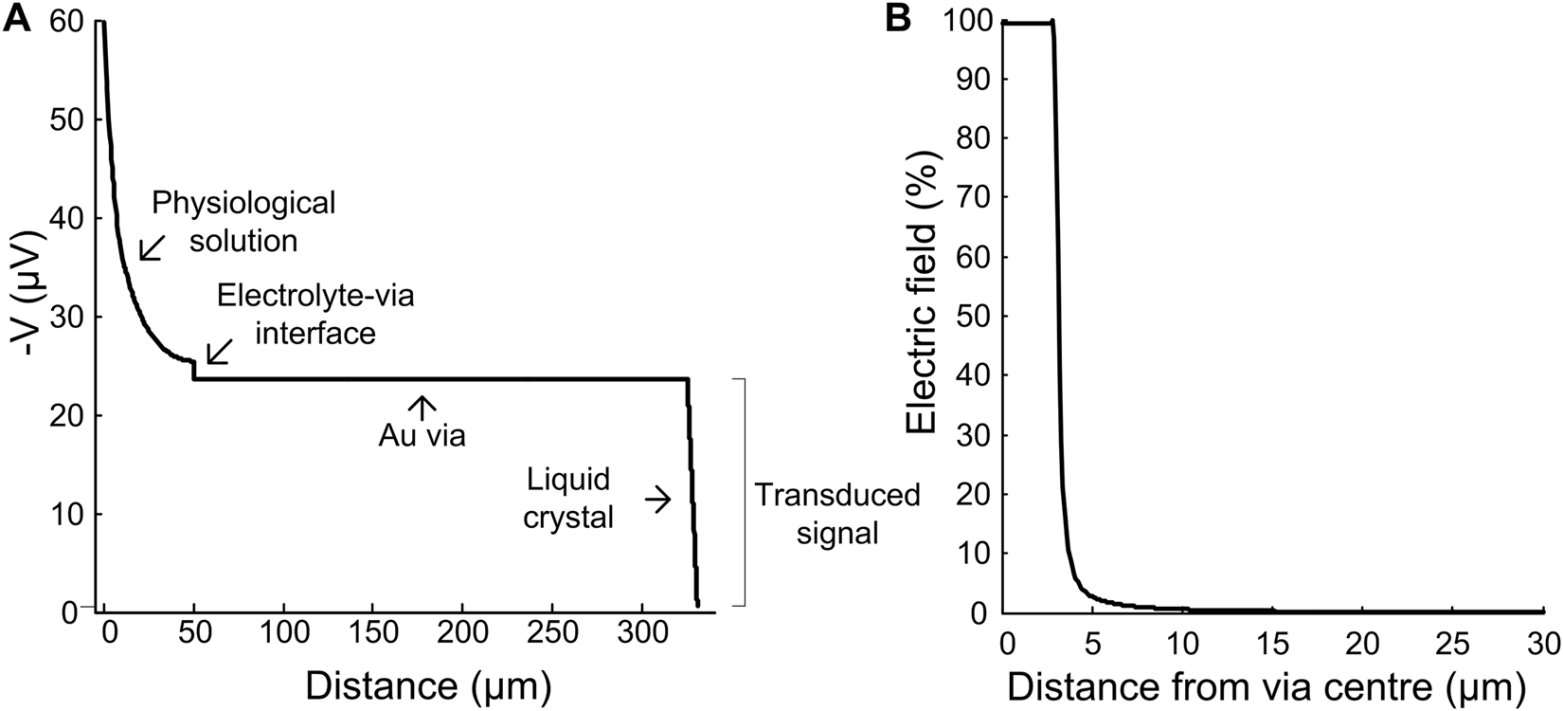
Assessing signal attenuation and channel leakage using a one channel simulation of the optrode device. (**A**) Signal attenuation from the source (neuron) through the various layers of physiological solution and optrode device. Note that the majority of the potential drop occurs in the physiological solution rather than across the optrode’s layers. (**B**) Electric field (*E*_*z*_) measured at middle of the LC layer along a line originating at a point directly underlying the centre of a via.

In Fig. 10 we plotted the biopotential drop across the various layers of the device. The voltage drop across the LC represents the transduced biopotential for read-out. The peak negative voltage at the via-LC interface is −24 *μ*V compared to −60 *μ*V at the neuron. Relative to the extracellular potential source, 95% of the total voltage attenuation occurred in the physiological solution, followed by 5% across the via-electrolyte bilayer.

#### Channel cross-talk

It is clear from Fig. 9 that changes in the polarisation of the LC are confined to LC regions directly below the vias. It can also be noted that changes in *P*_*Gz*_ occurred in channels not directly below the neuron. We attribute this to the spread of extracellular potential in physiological solution to vias further away from the neuron source, thus following multiple parallel channel paths to the ground electrode via the LC layer. This explains the reduction in the peak value of *P*_*Gz*_ at a given optrode when a multi-channel device is simulated compared to a single channel device.

Channel leakage was quantified in a single electrode simulation of the optrode device (Fig. 10). The electric field in the LC layer below the via dropped to less than 1% of its value 46 *μm* from the edge of the via.

To further quantitatively evaluate whether or not any cross-talk occurs between adjacent channels of the optrode device, the polarisation of the LC helical structure by the electric field, *P*_*Gz*_, was measured for the single-channel device and compared to the device with two vias, 150 *μm* centre-centre spacing apart. In both cases, the measurement was taken below the central via midway through the LC layer. The correlation coefficient between both signals was 0.9909 (*p* = 0), indicating no measurable interference between channels if numerical errors are considered.

#### Impedance and noise analysis

An important issue in the measurement of biopotentials is signal degradation due to weak coupling between cell and electrode and filtering by intermediate capacitive processes. Key to the analysis and appraisal of new MEA technology is quantification of the coupling efficiency based on a circuit representation of the device as shown in Fig. 2. Where traditionally this is in the form of a transfer function relating the impedance at the input of an electrical amplifier to that of the overall system, in the case of the proposed optrode array this is the ratio between LC and total impedances. The voltage sensed by the device may thus be defined as

39 $${V}_{LC}={V}_{bio}\frac{{Z}_{LC}}{{Z}_{total}}$$

where

40 $${Z}_{total}={R}_{sol}+{R}_{gsol}+{R}_{via}+{R}_{gW}+{Z}_{gB}+{Z}_{viaB}+\frac{{Z}_{stray}({Z}_{LC}+{R}_{ITO})}{{Z}_{LC}+{Z}_{stray}+{R}_{ITO}}\mathrm{.}$$

In our analysis we assume that *Z*_*stray*_ is non-existent, and the via (*R*_via_), ground wire (*R*_*gW*_) ground solution (*R*_gsol_) and ITO (*R*_ITO_) resistances are negligibly small, thence

41 $${Z}_{total}\approx {R}_{sol}+{Z}_{LC}+{Z}_{gB}+{Z}_{viaB}\mathrm{.}$$

The electrode-electrolyte bilayer impedance is one of the major sources of both signal attenuation as well as thermal noise in MEAs^35,36^, and remains a crucial parameter in the optimisation of electrical coupling between the biopotential-generating neuron or tissue and the device in extracellular space^2,6^. We have ${Z}_{viaB}={Z}_{CI}\parallel {Z}_{CT}$, where from equation (15) we obtain a specific capacitance of *C*_*viaB*_ = 0.6441 Fm^−2^ for a neuronal signal of amplitude 100 *μ*V, giving a bulk impedance *Z*_*CI*_ ≈ 10^5^ Ω at signal frequency 1 kHz, and from equation (17) a charge transfer impedance *Z*_*CT*_ ≈ 2.27 × 10^11^ Ω. Note that while the resulting interfacial impedance is quite high, $\sim 100$ kΩ at 1 kHz, the impedance of the LC layer, whose electrical behaviour may be approximated as that of a lossy capacitor having the complex Debye relative permittivity equation (21), is ideally about four to five orders of magnitude larger^37^, ensuring maximal biopotential drop across the LC. Our simulations show (Fig. 10) that attenuation in the case of ideal cell-optrode contact may amount to about 3% of signal amplitudes. Since the remaining significant term in equation (41) is *Z*_*viaB*_, and the LC impedance *Z*_*LC*_ seen from the via similarly scales inversely with optrode area, the coupling equation (39) is in fact largely independent of the size of the optrodes, an advantage over current MEA technology which imposes trade-offs between electrode spatial resolution and recording signal-to-noise ratio.

The major sources of noise in MEA recordings are electrical instrumentation followed by the electrochemical bilayer impedance, amounting to roughly 10 *μ*V overall^38^. Performing signal transduction and read-out in the optical domain has the advantage of greatly reducing the former, which is the larger of the two. In our system, noise arising from the ground bilayer $\sim 0.1$ *μ*V can be neglected. In order to quantify the thermal noise arising from the via bilayers, the Johnson-Nyquist noise power spectral density for our estimated impedance is

42 $$S(f)=4{k}_{B}T\,\Re \{{R}_{sol}+{Z}_{viaB}(f)\}=4{k}_{B}T({R}_{sol}+\frac{{R}_{CT}}{1+{\mathrm{(2}\pi {R}_{CT}{C}_{I})}^{2}{f}^{2}}),$$

where *R*_*CT*_ = *ρ*_*viaB*_/*A*, *C*_*I*_ = *C*_*viaB*_*A*, *k*_*B*_ is the Boltzmann constant, and the RMS voltage noise is given by

43 $${V}_{n}=\sqrt{{\int }_{{f}_{1}}^{{f}_{2}}S(f)\,df}\mathrm{.}$$

The integral in equation (43) evaluates to a sum of terms arising from the solution resistance 4*k*_*B*_*TR*_*sol*_Δ*f*, and from the surface impedance $\frac{2{k}_{B}T}{\pi {C}_{I}}\arctan \mathrm{[2}\pi {R}_{CT}{C}_{I}f{]|}_{{f}_{1}}^{{f}_{2}}$, where we assume a conservative maximal bandwidth Δ*f* = *f*_2_ − *f*_1_ of biopotential signals from *f*_1_ = 0.1 Hz to *f*_2_ = 20 kHz. This yields a predicted noise level of less than 2 *μ*V, or 2% of typical changes in extracellular neuronal potentials^6^.

### Optical analysis of device

The reflectance from the optrode as a function of time is shown in Fig. 8. We note that the optical signal exactly reproduces the polarisation signal. Both show a short delay of 15 *μ*s with respect to the electrical signal, due to the finite relaxation time of the LC, which is around 19 *μ*s.

**Figure 11:**
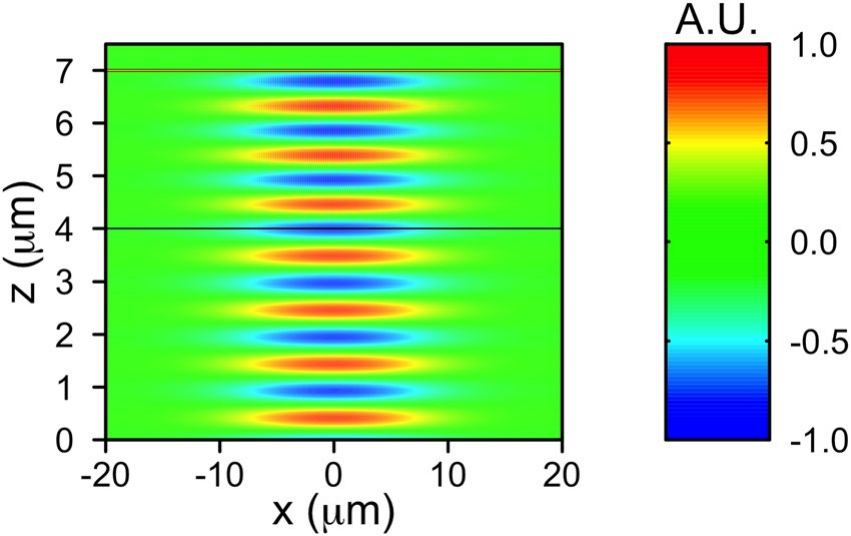
Electric field along the *y* axis, *E*_*y*_, of the Gaussian beam injected at *z* = 0 and propagating through the three regions of the device: glass (0 < *z* < 4 *μ*m), LC (4 < *z* < 7 *μ*m) and gold coated glass (7 < *z* < 7.5 *μ*m). The gold coating is at *z* = 7 *μ*m and the wavelength is *λ* = 1.55 *μ*m. *E*_*y*_ is plotted using arbitrary units (A.U.).

The electric field *E*_*y*_ in a typical simulation is reported in Fig. 11. It can be seen that the light is completely reflected by the gold layer and that the field at the boundaries *x* = ±20 *μ*m of the simulation domain is negligible, thus validating our approximations. From Fig. 11 we also observe the manner in which the LC diffracts the beam.

## Discussion

Theoretical analysis has been utilised extensively in the design of recording MEAs. In addition to product design, electric circuit models are instrumental in the quantitative analysis of electrode-electrolyte bilayers^22,39^. Finite element models of extracellular MEA recordings have been applied to investigate the effect of electrode size, position, and contact as well as to better understand the origin of local field potentials^7,40–45^.

These models have incorporated different levels of detail, from simply modelling a neuron and a recording electrode as a boundary with a potential drop^40,41^, adding an electrode-electrolyte bilayer^42,44,46^ and recording instrumentation input impedance^42,46^, and signal filtering during acquisition^40^.

We present a coupled electro-optical finite element model of an optrode device for sensing biopotentials, incorporating neuronal biophysics as well as the electro-optical transduction mechanism.

Our modelling strategy employed full coupling between volume conductor physics, neuron biophysics, and interface bilayers. This approach eliminates the need for a transformation matrix to map the output of neuronal simulations conducted in one software onto the MEA finite element model in a second software^40,41,43,44,46^.

Simulations indicate minimal electrical cross-talk between channels in the optrode array. From the optical perspective, the use of Gaussian light beams with diameter smaller than via diameter (60 *μ*m) will ensure no optical cross-talk either. Hence in terms of the overall transduction mechanism, our design ensures that no interference occurs between biopotential signals sensed at adjacent channels leading to better spatial resolution.

Our theoretical analysis of the optrode’s temporal response predicts an electro-optical transduction delay of approximately 15 *μ*s, attributed to the time constant of the Debye relaxation of the LC. As the bandwidth is limited by the LC response time, this suggests that our technology can faithfully acquire frequencies up to 33 kHz (Nyquist rate), well within the range of biopotential signals. In fact, the response time constant of the LC can be considered an advantage, in that it offers inherent low-pass filtering. The temporal sampling will be handled by photo-detectors with sufficiently larger bandwidth than those of bioelectric signals.

Thermal noise, generated by random motion of charge carriers in materials, is a critical consideration when analysing recording microelectrodes. However, there is considerable variability in the literature on what contributes to thermal noise. For example, Rocha *et al*.^38^ considered the electrode-electrolyte interface alone, whereas Lempka *et al*.^40^ also included tissue and cell membrane impedances as well as the shunt capacitance of the electrode shank. Reasonable simplifications to the impedances represented in the circuit model in Fig. 2 allowed us to discount all sources except for the bilayers in our estimate. We predict an optrode-electrolyte bilayer thermal noise level noise level of less than 2 *μ*V for the 0.1 Hz to 20 kHz bandwidth. The acquisition bandwidth itself may be limited to improve the signal to noise ratio^40^.

The 60 *μ*m diameter of vias in our simulated optrode device is within the range found in available MEAs^11^. Our theoretical analysis indicates that electro-optical coupling is largely independent of the size of the optrodes, and the via diameter could be reduced without compromising recording signal-to-noise ratio, a drawback of traditional MEAs. Signal amplifiers in electrophysiological acquisition systems utilising traditional electrodes (glass microelectrodes or metal electrodes) need to be engineered such that their input impedance compensates for the increase in electrode impedance as the electrode size is reduced. Theoretical analysis indicates that our optrode design and fabrication method alleviates the need for this size-specific configuration; the transducer impedance depends on size of the via and hence the ratio of electrode impedance and transducer impedance is constant. Hence future devices will be fabricated with smaller vias.

Optical imaging of tissue excitation can be achieved using voltage-sensitive dyes, which change fluorescence in response to changes in transmembrane potential. This has been applied in neuronal (e.g^47^.) as well as cardiac tissue preparations^48^. However, their use is limited by their release of free radicals leading to cellular death, which prevents prolonged observations.

### Device sensitivity

In practice, the optrode device sensitivity could be improved by several approaches.

In our simulation, most of the signal attenuation (95%) occurred across the 50 *μ*m physiological solution between neuron and via-electrolyte surface (Fig. 10). This is consistent with the separation used by Lempka *et al*.^40^ in their theoretical analysis of intracortical microelectrode recording, although conservative compared to the 0–200 nm range used by Joye *et al*.^39^ in their circuit model of the cell-electrode interface in MEAs. In practice, closer proximity between studied cells and vias can be ensured during experiments via mechanical (slice holders, 3D protruding electrodes) or chemical (e.g. poly-L-lysine) means. As the extracellular potential drops inversely with distance from source, optimising tissue-electrode contact can result in a significant sensitivity increase.

Whilst our simulations demonstrated the feasibility of the device to detect extracellular potentials from sub-cellular regions of a single neuron, the field potentials from aggregates of neurons could be as high as 100 *μ*V, even reaching 1 mV in cardiac muscle. This would result in a 15-fold increase in signal strength compared to the 60 *μ*V from a single simulated neuron.

A number of physical systems can be employed for optical readout of reflectance such as an optic fibre with light source and detector, or fluorescent microscopy systems. For the former, the major cause of noise in the optical readout system, as described for instance by Brodzeli *et al.*^16^, is the light source. Sources with a relative intensity noise (RIN) of around −140 dB/Hz at high frequencies have been demonstrated^49^. This corresponds to an RMS value of 6 ppm for the RIN integrated over a 4 kHz bandwidth. Such a low noise floor would allow detection of the peak simulated in this paper. Please note that in the approach presented in this paper the read-out opto-electronics is completely decoupled from the electrical part of the optrode (it could even be in a different location kilometres away) and it may be made more complex without affecting the electrical coupling between the optrode and the biological specimen.

On the other hand, if microscopy systems are employed for the optical readout, the signal to noise ratio will depend on the quantum efficiency, read noise and output resolution of photomultiplier tube or camera systems.

Finally, LCs with stronger optical response are available and would further improve the signal-to-noise ratio.

We point out that the above discussion focuses on real time measurements and completely neglects additional signal processing. As an example, we have already demonstrated an equivalent noise floor of 8 *μ*V in spectral measurements with 1 second acquisition time.

### Model limitations

Setting up simulations involves a trade-off between accuracy and computational requirements. In multiphysics models such as the one we present here, compromises have to be made in terms of accuracy of representation of certain physics. We have focused on accurate mathematical representation of all the physical components of the electro-optical transduction mechanism, as our aim was to inform and optimise the design of our optrode device. On the other hand our requirements for the neuronal physics were to generate a basic extracellular potential signal without necessarily enforcing strict physiological and anatomical accuracy. In addition, we simulated the device’s performance under ideal conditions, excluding ambient electromagnetic noise from our analysis.

We employed a simplified geometrical representation of a neuron, rather than an anatomical reconstruction. While the effect of geometry on local field potentials cannot be discounted, we postulate that for our simulation scenario (single unit, no synaptic input, impulse initiated retrogradely from the axon), neuronal biophysics will have a greater impact on the strength of the extracellular signal compared to the neuronal geometry. The length of the limited number of branches in our model could be considered a lumped representation of the extensive dendritic branching of neurons. In addition, the model is limited by the fact that we assumed uniform biophysical properties along the neuron, where in fact inhomogeneity of ion channel expression, therefore conductances, is well established in different segments of the one neuron.

In terms of device modeling we assumed that the shunt impedance *Z*_*stray*_ is infinitely large, thereby ignoring the Si substrate-via and Si substrate-electrolyte bilayers as well as fringing fields. In practice, Si_3_N_4_ and SiO_2_ are applied as protective layers between the gold electrodes and Si substrate, effectively eliminating the stray conductance. However, we cannot rule out the presence of shunt capacitance. In their finite-element analysis Cantrell *et al*.^20^ reported significant shunt capacitance through the insulator-electrolyte interface. However, in comparison to their simulated Levick-style electrodes, in which the insulation (1 *μ*m) was thinner than the average radius of the electrode metal, the thickness of the Si substrate (275 *μ*m) in our device is larger than the via-edge to via-edge distance (90 *μ*m), and equal to the electrolyte to LC distance. Therefore, we can expect a significantly smaller shunt capacitance.

## Conclusion and future work

Towards developing a biopotential optrode array we present an *in silico* analysis of the device’s performance. The electro-optical transducer is a liquid crystal layer, which offers the advantages of electrically decoupling the biological signal from the acquisition hardware and of achieving an efficient neuron-optrode electrical coupling that is largely independent of the size of the electrodes. Theoretical analysis confirms the capability for high-resolution spatio-temporal imaging of excitable tissue activity, predicts no cross talk between channels, and indicates that the transducer impedance automatically scales with the electrode bilayer impedance and is large by comparison, therefore overcoming some of the main limitations of standard MEAs. The photonic nature of the sensor technology ensures that it is immune to the wiring and packaging limitations associated with increased channel density in traditional MEAs. The *in silico* analysis presented in this paper was instrumental to the design and fabrication of prototype devices. Future work will include bench-top physical characterisation followed by testing using biological samples, and will be reported in subsequent manuscripts.

## Electronic supplementary material

Supplementary Information
