# Supplementary material for: A biopotential optrode array: operation principles and simulations
Source: Sci Rep. 2018 Feb 9;8:2690. doi: 10.1038/s41598-018-20182-x (PMC5807498; doi:10.1038/s41598-018-20182-x)
Supplement: Supplementary file 1 — Supplementary Information [file 41598_2018_20182_MOESM1_ESM.pdf]

A BIOPOTENTIAL OPTRODE ARRAY: OPERATION PRINCIPLES AND SIMULATIONS

SCIENTIFIC REPORTS

Supplementary Infromation

Amr Al Abed[1,*], *Hrishikesh Srinivas*[1,2], *Josiah Firth*[2], *François Ladouceur*[2], *Nigel H. Lovell*[1] and *Leonardo Silvestri*[1,2]

[1]Graduate School of Biomedical Engineering, UNSW, Australia
[2]School of Electrical Engineering and Telecommunications, UNSW, Australia
* Corresponding author: Email amra@unsw.edu.au

January 24, 2018

Parameters

Table 1: Model parameters.

Parameter	Value (units)	Explanation
Neuron biophysics		
r	2.5 (μm)	Radius of neuron cable
σ_i	1.67 (Sm^{-1})	Intracellular conductivity
C_m	1 (μFcm^{-2})	Specific membrane capacitance
$E_{r,Na}$	55 (mV)	Reversal potential of sodium current
$E_{r,K}$	-72 (mV)	Reversal potential of potassium current
$E_{r,L}$	-60 (mV)	Reversal potential of leakage current
$\bar{g}_{Na}$	120 (mScm^{-2})	Maximum conductance of sodium current
$\bar{g}_K$	36 (mScm^{-2})	Maximum conductance of potassium current
$\bar{g}_L$	0.3 (mScm^{-2})	Maximum conductance of leakage current
Electrical physics		
σ_{sol}	1.6048 (Sm^{-1})	Electric conductivity of 0.154 M (0.9% w/v) NaCl at 25°C [1]
σ_{Au}	58.1×10^6 (Sm^{-1})	Electric conductivity of gold vias
$\sigma_{silicon}$	10^{-12} (Sm^{-1})	Electric conductivity of silicon substrate
$\epsilon_{r,sol}$	99	Relative permittivity of physiological solution[1] [2]
$\epsilon_{r,Au}$	1	Relative permittivity of gold vias [3]
$\epsilon_{r,silicon}$	11.7	Relative permittivity of Si substrate
R_{gW}	10 Ω	Resistance of cable connecting the ITO and ground electrode
Liquid Crystal		
d	3 (μm)	LC cell thickness
θ	32 (°)	Tilt angle [4]
p_0	200 (nm)	Helical pitch [4]
E_c	1.56 ($\text{V}\mu\text{m}^{-1}$)	Critical electric field [5]
ϵ_0	8.854×10^{-12} (Fm^{-1})	Vacuum permittivity
τ_c	19.3 (μs)	Characteristic relaxation time of the LC [6]
ϵ_s	33.3	Static LC permittivity [6]
ϵ_∞	3.5	High frequency LC permittivity [6]
n_o	1.5	Ordinary refractive index of LC [5]
n_e	1.72	Extraordinary refractive index of LC [5]
ϵ''	0.01 i	Imaginary part of the LC dielectric constant
Optical		
n_g	1.52	Refractive index of glass
λ	1.55 (μm)	Wavelength of light
β	30 (°)	Angle between the incident linear polarisation and the polariser
w_0	10 (μm)	Beam waist radius
z_0	7 (μm)	Beam waist position
Bilayer physics		
d_{OHP}	5 (Å)	Distance from outer Helmholtz plane to via metal [7]
ϵ_0	8.854×10^{-12} (Fm^{-1})	Permittivity of free space
z	1	Number of electrons involved in interfacial gold redox
e	1.602×10^{-19} (C)	Electron charge
U_t	0.0259 (V)	Thermal voltage at room temperature
n^0	$154 N_A$ (m^{-3})	Bulk number density of ions in 0.154 M (0.9% w/v) NaCl

[1] Based on body fluid estimate at 10 Hz

Numerical solvers

The time-dependent finite element models were solved using the PARDISO direct fully-coupled solver. Model results were outputted at 20 kHz. An adaptive backward differential formula time stepping routine was used with maximum order 5, absolute tolerance 0.001 and relative tolerance 0.01.

Meshing

Different meshes were used for Stages 1 and 3 of the modelling pipeline (Fig. 3). Stage 1 models were meshed using a mix of edge, triangular, quadrilateral, tetrahedral, and prism element types. For the temporal response analysis (Fig. 7), the elements of the neuron were set to have a maximum length of 25 μm. The edges of vias and via-electrolyte boundaries had a maximum element size of 2.5 μm, and the LC and via/silicon layers swept with 8 and 35 elements, respectively. For the spatial response analysis, the elements of the neuron were set to have a maximum length of 25 μm. The edges of vias and via-electrolyte boundaries had a maximum element size of 25 μm, and the LC and via/silicon layers swept with 3 elements each.

Stage 3 models were meshed using rectangular elements with maximum edge sizes 75 nm in the z direction and 10 nm in the x direction. This choice ensures that there are at list 20 points per wavelength in the z direction and 20 points per helix pitch in the x direction.

Quadratic Lagrange or quadratic discontinuous basis functions were used for all FEA simulations.

Transition boundary condition

In COMSOL, the transition boundary condition is used to model layers that are geometrically, but not electrically, thin. It represents a discontinuity in the tangential electric field between two regions, indicated by the subscripts 1 and 2, described by the relations:

$$\mathbf{J}_{s1} = \frac{Z_S\mathbf{E}_{t1} - Z_T\mathbf{E}_{t2}}{Z_S^2 - Z_T^2} \quad (1)$$

$$\mathbf{J}_{s2} = \frac{Z_S\mathbf{E}_{t2} - Z_T\mathbf{E}_{t1}}{Z_S^2 - Z_T^2} \quad (2)$$

$$Z_S = \frac{-i\omega\mu}{k}\frac{1}{\tan(kd)} \quad (3)$$

$$Z_T = \frac{-i\omega\mu}{k}\frac{1}{\sin(kd)} \quad (4)$$

$$k = \omega\sqrt{(\epsilon + (\sigma/(i\omega)))\mu}, \quad (5)$$

where $\mathbf{J}_s$ is the induced surface current density and ϵ, σ, μ are the permittivity, electrical conductivity and permeability of the layer's material, respectively. In

our simulations we used $\epsilon = -96.957 + 11.504i$ [8], $\sigma = 0$, $\mu = 1$ and $\omega = 2\pi c/\lambda$, where c is the speed of light, $\lambda = 1.55$ μm.

Bibliography

[1] AP Mazzoleni, BF Sisken, and RL Kahler. Conductivity values of tissue culture medium from 20 degrees c to 40 degrees c. *Bioelectromagnetics*, 7: 95–99, 1986.

[2] D Andreuccetti, R Fossi, and C Petrucci. An internet resource for the calculation of the dielectric properties of body tissues in the frequency range 10 hz - 100 ghz. based on data published by c.gabriel et al. in 1996. `http://www.niremf.ifac.cnr.it/tissprop`, 1997.

[3] J Lourtioz, H Benisty, V Berger, J Gerard, and D Maystre. *Photonic Crystals: Towards Nanoscale Photonic Devices*. Springer-Verlag Berlin and Heidelberg Gmbh 2005.

[4] Z Brodzeli, L Silvestri, A Michie, Q Guo, EP Pozhidaev, V Chigrinov, and F Ladouceur. Reflective mode of deformed-helix ferroelectric liquid crystal cells for sensing applications. *Liquid Crystals*, 40:1427–1435, 2013. doi: 10.1080/02678292.2013.807942.

[5] AD Kiselev, EP Pozhidaev, VG Chigrinov, and H Kwok. Polarization-gratings approach to deformed-helix ferroelectric liquid crystals with subwavelength pitch. *Phys. Rev. E*, 83:031703, 2011. doi: 10.1103/PhysRevE.83.031703.

[6] H Srinivas, A Al Abed, F Ladouceur, NH Lovell, and L Silvestri. Modeling the Debye dielectric response in the time domain for a liquid crystal-based biopotential optrode. In *Conf Proc IEEE Eng Med Biol Soc. 2016*, pages 4857–4860, 2016. doi: 10.1109/EMBC.2016.7591815.

[7] GTA Kovacs. Introduction to the theory, design, and modeling of thin-film microelectrodes for neural interfaces. In DA Stenger and TM McKenna, editors, *Enabling Technologies for Cultured Neural Networks*, pages 121–165. Academic Press, London, U.K, 1994.

[8] AD Rakić, AB Djurišić, JM Elazar, and ML Majewski. Optical properties of metallic films for vertical-cavity optoelectronic devices. *Appl. Opt.*, 37: 5271–5283, 1998. doi: 10.1364/AO.37.005271.
